# Anterior Spinal Artery Syndrome Following Coronary Artery Bypass Grafting: a Case Report

**DOI:** 10.21470/1678-9741-2016-0039

**Published:** 2017

**Authors:** Seyed Mohsen Mirhosseini, Soheil Meghdadi, Ali Sanjari Moghaddam

**Affiliations:** 1 Cardiovascular Research Center, Shahid Beheshti University of Medical Sciences, Tehran, Iran.; 2 Department of Neurology, Moheb Mehr Hospital, Tehran, Iran.; 3 School of Medicine, Shahid Beheshti University of Medical Sciences, Tehran, Iran.

**Keywords:** Anterior Spinal Artery Syndrome, Coronary Artery Bypass, Paraplegia, Postoperative Complications

## Abstract

We present a patient with unstable angina candidate for coronary artery bypass grafting. Saphenous vein graft was used in obtuse marginal and left internal mammary artery to left anterior descending artery properly. After surgery, the patient experienced flaccid paralysis of lower limb and impaired sensation of touch and warmth of knee and below. A computed tomography angiogram of lower limbs and thoracolumbar magnetic resonance imaging showed no abnormality. Based on the symptom, clinical diagnosis of anterior spinal artery syndrome was considered. The artery of Adamkiewicz is an important supplier to the anterior spinal artery. Internal thoracic mammary artery, used in coronary artery bypass grafting, is suspected as a collateral supplier of the artery of Adamkiewicz and has been accused for cause of spinal infarction.

**Table:** 

Abbreviations, acronyms & symbols	
CABG	= Coronary artery bypass grafting
CPB	= Cardiopulmonary bypass
CT	= Computed tomography
IABP	= Intra-aortic balloon pump
MRI	= Magnetic resonance imaging

## INTRODUCTION

Neurological complications after coronary artery bypass grafting (CABG) are usually severe^[1]^. Paraplegia is a very rare complication of CABG. Post cardiac surgery paraplegia is due to spinal cord ischemia or infarction^[2]^.

A case of paraplegia after CABG is reported, a clinical diagnosis of anterior spinal artery syndrome.

## CASE REPORT

The patient was a 61-year-old heavy smoker man, admitted to Moheb hospital with unstable angina. He had history of type 2 diabetes mellitus, chronic obstructive pulmonary disease and hypertension. The patient had been administrating antihypertensive agents, bronchodilators and insulin for diabetes.

Laboratory findings were just remarkable for mild normochromic normocytic anemia. A coronary angiogram showed a significant three-vessel disease and major stenosis of left main artery. In addition, a color-Doppler sonography of the carotid arteries displayed echogenic plaque causing moderate stenosis of both carotids. Patient was considered for emergent CABG.

After general anesthesia, cardiopulmonary bypass (CPB) was performed. Manual examination of aorta revealed no atherosclerotic plaque. Surgery consisted of saphenous vein graft in obtuse marginal and left internal mammary artery to left anterior descending artery. Right coronary artery was not eligible for graft. The clamp time of aorta was 30 minutes and the patient was weaned easily from the CPB with no need of inotropic drugs. He was transferred to intensive care unit and extubated a few hours later.

On post-operative day one, patient was too drowsy to examine but obvious movement of his lower limbs was seen. In the morning of second postoperative day, a full assessment revealed flaccid paralysis and lack of patellar reflex of both lower limbs (muscle strength grade zero) and impaired sensation of touch and warmth of knee and below. However, proprioceptive sensation and vibration were intact. Cranial nerve function and mental status were normal. No upper limb weakness was detected and pulses were palpable and symmetric at the peripheries. To exclude vascular pathology, a color-Doppler sonography of lower limbs was performed and showed a normal blood flow of arteries and veins. A computed tomography (CT) angiogram of lower limbs and thoracoabdominal region revealed multiple atherosclerotic plaques, but no embolism. In addition, magnetic resonance imaging (MRI) of thoracic spine depicted no abnormality. A clinical diagnosis anterior spinal artery syndrome was carried out, and the patient was discharged one week later, scheduled for rehabilitation. One year after the follow-up period, no improvement of motor function was observed. The patient got bed sore ensuing immobility and developed infectious diabetic foot due to poor controlled hyperglycemia. Finally, died of sepsis complications and multiorgan failure.

## DISCUSSION

Paraplegia following heart and aorta interventions and surgery is an uncommon complication. However, the rate of spinal cord damage is reported to be 5-10% after dissecting aneurysm and 5-80% in non-stenotic disease of the descending aorta^[3]^. Paraplegia after CABG is a very rare condition and just a small number of reports are available^[1,4-7]^. Spinal cord ischemia or infarction seems the most probable causes^[2]^. The reason of ischemia and infarction is still speculative. Risk factors of spinal cord infarction include perioperative or intraoperative hypotension, use of an intra-aortic balloon pump (IABP), manipulation of the aorta, and medulla collateral circulation blockage and spinal cord lesions (such as intervertebral disk herniation and iatrogenic injuries during regional anesthetic blockage)^[1]^. We used general anesthesia for operation and aorta was manipulated just for manual examination of grafting areas. In addition, patient experienced no episode of hypotension and no need for IABP. Our patient had no major risk factor for spinal infarction, but with regard history of long-term diabetes and hypertension, he was predisposed to peripheral artery diseases. Nevertheless, embolism of main branch of spinal cord arteries and lower limbs arteries were excluded by a normal CT angiogram of lower limbs and thoracoabdominal region. However, the thoracolumbar MRI was normal, but observation of infarction of watershed area of spinal cord is usually difficult and often high resolution MRI is required. In this study, patient's feature had most consistency to anterior spinal artery syndrome and normal MRI just rule out other possibilities. The anterior spinal artery and two small posterior arteries derived from vertebral artery and make up the three longitudinal vessels^[8]^. The anterior spinal artery supplies the frontal two thirds of the spinal cord^[2]^. The artery of Adamkiewicz is a medullary feeder artery, which fortifies blood supply of three longitudinal vessels and is an important supplier to the anterior spinal artery and the lumbar region of the spinal cord^[2]^. Adamkiewicz supplies the T9 to L2 spinal levels. Internal thoracic mammary artery, used in CABG, is considered as a collateral supplier of the artery of Adamkiewicz and has been accused for cause of spinal infarction in some papers^[1,2,9]^. Studies established few ways to reduce risk of postoperative paraplegia, including perioperative cerebrospinal fluid drainage^[10]^, local hypothermia^[11]^ and steroids^[12]^, but still there is no unanimity regarding the best method to decreased incidence of postoperative paraplegia.

In the present study, we made the hypothesis that internal mammary artery which is grafted to left main coronary artery supplies the artery of Adamkiewicz as a collateral circulation of spinal cord.

**Table:** 

Authors' roles & responsibilities	
SMM	Conception and study design; manuscript redaction or critical review of its content; final manuscript approval
SM	Conception and study design; manuscript redaction or critical review of its content; final manuscript approval
ASM	Conception and study design; manuscript redaction or critical review of its content; final manuscript approval
